# The Probationer's Course

**Published:** 1907-08-03

**Authors:** 


### Nursing Administration.

### THE PROBATIONER'S COURSE.

It is a truism that the value of the probationer's training depends in large measure on the extent of the opportunities afforded her for gaining experience. It is not so clearly understood, however, that these opportunities depend less on the size of the hospital than on the system of the matron. It is in the power of the matron to minimise the advantages which are presumably offered in a large institution, by limiting the work of the probationers to certain departments only, by keeping slow pupils at mechanical work, and by making use of the quick and eager ones to the detriment of their real grounding in the principles and practice of nursing.

Superintendents who have had the advantage of experience in both large and small hospitals are unanimously agreed that the soundest training can be afforded to probationers in hospitals of the ordinary medium-sized provincial type, which, by their simplicity of structure, make it possible to pass all probationers through each department in the course of their three years' training. Such hospitals contain, as a rule, five principal wards, two surgical wards (male and female), and two medical wards (male and female), with a ward for children. In addition, there will be an isolation ward, and several smaller ones for special cases. In a hospital of this description the usual rule is for each probationer to pass on in rotation, taking three months' day duty and three months' night duty in each principal ward. This disposes of two and a-half years of her training, the remainder being filled in with special cases, and theatre or out-patients' work. It will hardly be practicable, even under this simple system, to afford each probationer during her term experience of theatre work, but the matron can generally arrange to give experience among the out-patients to those who miss the theatre, and in this department it is now usual to perform many minor operations, which do, to a certain extent, make up for the loss of experience in the weightier responsibilities of the theatre. It is not every probationer who is fit during her years of training to be of use in the theatre. A nurse who has passed through every department of a moderate-sized hospital in this manner is certainly in a very good position as regards her future, although she may perhaps have had no opportunity of seeing out-of-the-way cases such as may fall in the day's routine as a matter of course to the probationer in a big clinical hospital. The duties of the matron as regards organising the course of her probationers in such an institution is greatly simplified, and many perplexities which beset the distribution of the work in a large institution are avoided altogether.

The plan of keeping each probationer three months on day and three months on night duty, generally in the same ward, prevails also in many hospitals of such a size that it is impracticable to give each nurse experience in each ward. At St. George's Hospital the ordinary practice is to retain the probationer six months on alternate day and night duty in the same ward, thus affording her during her three years six changes. At the end of every three months the matron receives a report from the sister under whom the probationer has been working, stating what has been learned during her stay in the particular ward, and a glance at these reports enables the matron to ascertain whether there have been any gaps in the training and to take steps to rectify omissions. The probationers get no theatre work until they have been at least a year under training. In their second year they serve, as opportunity offers, one month in the theatre as junior probationer; and in their third year they get, if possible, the opportunity of serving there for another month as senior probationer. In the fourth year they are staff nurses.

At Guy's Hospital it is usual for probationers to be changed every month during their first year, and after that they remain one year in a given department.

At most Poor-law infirmaries the practice is for probationers to remain three months in each ward.

In a clinical hospital where there is a great deal of life and movement, and where, as a rule, discipline is easy to maintain, the probationer can with advantage remain longer in one ward than in a smaller institution where, unless moved at comparatively frequent intervals there is some danger of her falling into a routine bad for herself and bad for her work. If it is thought desirable to give frequent changes it should be at the beginning of the work, when all is new and the work which falls to the lot of the probationer is purely mechanical in nature. It is a good plan, at any rate during the time which is spent on trial, before the probationer is regularly engaged, to allow her to work under more than one sister, so that her qualities may be justly gauged. Some sisters are of so gentle and tactful a disposition that no one can fail to do at least passably under them, and bad characteristics fade into insignificance in their presence. Yet it will not do to engage women who are perhaps incapable of doing their work unless very gently handled, at the recommendation of such gifted teachers. On the other hand, and far more frequently met with, are the sisters who take a dark view of human nature, and are given to unreasonable dislikes. Thus it is better to get two or three verdicts before deciding for or against the new pupil. A great deal can be done by good organisation to equalise the training, and give additional advantages to probationers who seem to have come short. But to this end the matron must know exactly what to expect from the time spent in each department, and study to keep the balance even between her duty to the probationer and her duty to the institution.

Both in large and small institutions incessant vigilance is needed if to ensure that no important hiatus ensues in the experience of nurses sent out with the hall-mark of a certificate of training. Undoubtedly much depends on the diligence of the nurse herself, but it is on the matron in the last resort that the full responsibility must rest, and it is for her not merely to provide opportunities for instruction, but also to guard against the possibility of such opportunities having been wasted.

